# Fifteen-year trends in diabetes drug management and control in French-speaking Switzerland

**DOI:** 10.1186/s13098-025-01620-z

**Published:** 2025-02-12

**Authors:** Ariane Pauli, Abdullah Alkandari, Pedro Marques-Vidal

**Affiliations:** 1 Doctoral School of the Faculty of Biology and Medicine, University of Lausanne, Lausanne, Switzerland; 2 Kuwait Institute for Scientific Research, Kuwait City, Kuwait; 3 Department of medicine, internal medicine, Lausanne university hospital, 46 rue du Bugnon, Lausanne, 1011 Switzerland

**Keywords:** Diabetes, Antidiabetic treatment, Glycaemic control, Switzerland

## Abstract

**Objective:**

Drug management of type 2 diabetes (T2D) should comply with established guidelines. Still, little is known about how drug management of T2D in Switzerland has evolved over time. We aimed at assessing 15-year trends in antidiabetic drug prescription and its effectiveness in reducing fasting plasma glucose (FPG) levels.

**Research design and methods:**

Data from the baseline (2003–2006) and three follow-ups (2009–2012, 2014–2017 and 2018–2021) of a population-based study conducted in Lausanne, Switzerland. Participants treated for T2D were included. At baseline and the follow-ups, participants had their antidiabetic drugs collected, together with their FPG and glycated haemoglobin (HbA_1_c) levels.

**Results:**

There were 274, 280, 268 and 195 participants treated for T2D at the baseline, first, second and third follow-ups, respectively, of whom 101 (36.9%), 103 (36.8%), 138 (51.5%) and 84 (43.1%) were controlled (FPG < 7 mmol/L). During the study period, the percentage of biguanides remained stable, the percentage of sulfonylureas and thiazolidinediones decreased, and the percentage of SGLT2 and DPP4 inhibitors increased, but no consistent association with T2D control was found. On bivariate and multivariable analysis, participants with newly diagnosed T2D had a higher likelihood of being controlled than participants with established T2D: odds ratio (95% CI) 3.39 (1.89–6.07), 5.41 (2.25-13.0) and 3.47 (1.45–8.31) for the first, second and third follow-ups on multivariable analysis, respectively.

**Conclusions:**

Despite the prescription of novel antidiabetic drugs, half of participants treated for diabetes do not achieve adequate control in Switzerland. Participants with newly diagnosed diabetes achieve much better control than participants with established diabetes.

**Supplementary Information:**

The online version contains supplementary material available at 10.1186/s13098-025-01620-z.

## Introduction

Prevalence of diabetes, namely type 2 diabetes (T2D) is increasing worldwide [1]. In Switzerland and other European countries, diabetes management is still subject to improvement, as almost half of treated patients with diabetes are inadequately controlled [2–4]. Diabetes treatment is based on a multitude of approaches including lifestyle modifications and antidiabetic drug prescription [5]. Diabetes medication has changed over the years; new drugs have become available, while others are used less frequently for economic and safety reasons. Most national and international guidelines propose metformin as a first-line drug treatment for T2D [6, 7]. Combining metformin with sodium-glucose transporter 2 (SGLT2) inhibitors or glucagon-like 1 peptide (GLP1) analogues is also recommended [6]. Still, little is known about how T2D drug management in Switzerland has developed over time and whether these changes have led to a better control of glycated haemoglobin (HbA_1_c) or glucose levels.

Evaluating clinical practices in the management of T2D can significantly impact healthcare expenditures and improve patient outcomes by identifying areas for efficiency and optimization in treatment protocols. By evaluating the (cost-)effectiveness of various interventions, clinicians and policymakers can prioritize evidence-based practices that reduce unnecessary hospitalizations, complications, and long-term healthcare costs associated with poorly managed T2D. For example, early intervention strategies, better glycaemic control, and comprehensive care models can prevent progression of the disease and associated comorbidities, leading to fewer emergency room visits and less need for costly treatments. In addition, such assessments can guide healthcare systems in directing resources to the most effective therapies, ultimately improving patients’ quality of life, reducing disparities in care, and maximizing the sustainability of healthcare systems.

We thus assessed the evolution of antidiabetic drug prescription in a sample of the French-speaking Swiss population, and their association with adequate control of HbA_1_c or fasting plasma glucose (FPG) levels. We also assessed whether newly diagnosed cases of T2D would benefit from the newest antidiabetic drugs. Our hypotheses were that (1) the prescription rates of the new antidiabetic drugs such as DPP4, GLP1 analogues and SGLT2-inhibitors increased, (2) participants treated with the new drugs would achieve a better control of HbA_1_c or glucose levels than participants who do not receive them, and (3) participants with newly diagnosed T2D would benefit more frequently from the new antidiabetic drugs than participants with established T2D.

## Methods

### Participants

We used data from the CoLaus study, a prospective, population-based study conducted in Lausanne, Switzerland. The objectives and characteristics of the CoLaus study have been reported previously [8]. Briefly, CoLaus is an ongoing prospective study aiming to assess the determinants of cardiovascular and psychiatric diseases using a population-based sample drawn from the city of Lausanne, Switzerland. The baseline study was conducted between June 2003 and May 2006; the first follow-up was performed between April 2009 and September 2012; the second follow-up was performed between May 2014 and April 2017 and the third follow-up was performed between April 2018 and May 2021. At baseline and follow-ups, participants responded to several questionnaires, underwent a physical examination, and had blood drawn for analyses.

### Diabetes

All prescribed and non-prescribed medications were collected and coded according to the Anatomical Therapeutic Chemical (ATC) classification of the World Health Organization (WHO). The ATC codes corresponding to the different categories of antidiabetic drugs are indicated in supplementary Table 1. Combinations of antidiabetic drugs were further split into the different antidiabetic drug classes.

Blood was drawn in the fasting state and biological assays were performed by the Lausanne University Hospital (CHUV) Clinical Laboratory on fresh blood samples within 2 h of blood collection. FPG was assessed by glucose hexokinase. In the second and third follow-ups, HbA_1_c levels were also measured by high performance liquid chromatography using Bio-Rad, D-10TM system. For the main analysis, diabetes was defined as a FPG ≥ 7 mmol/L or presence of antidiabetic drug treatment. Diabetes control was defined as a FPG < 7 mmol/L [9]. Incident T2D was assessed at follow-ups and defined as a participant devoid of T2D in the previous survey and who developed the condition in the following one.

### Covariates

Participants were queried regarding their lifestyle and socio-economic status. Educational level was self-reported using a questionnaire and categorized into low (mandatory or apprenticeship), middle (high school) and high (university). Educational level served as a proxy for socio-economic status. Smoking status was self-reported and categorized into never, former, and current. Participants were asked if they were on an antidiabetic or on any type of diet and, for the first and second follow-ups, were invited to fill a questionnaire assessing physical activity. Sedentary status was defined as spending more than 90% of the daily energy in activities below moderate- and high-intensity [10].

Body weight and height were measured with participants barefoot and in light indoor clothes. Body weight was measured in kilograms to the nearest 100 g using a Seca^®^ scale (Hamburg, Germany). Height was measured to the nearest 5 mm using a Seca^®^ (Hamburg, Germany) height gauge. Body mass index (BMI) was computed, and participants were further categorized into normal (BMI < 25 kg/m^2^), overweight (BMI ≥ 25 and < 30 kg/m^2^) and obese (BMI ≥ 30 kg/m^2^).

Waist circumference was measured mid-way between the lowest rib and the iliac crest using a non-stretchable tape and the average of two measurements was taken. Abdominal obesity was defined as a waist circumference > 102 cm (men) or > 88 cm (women).

Blood pressure (BP) was measured three times using an Omron^®^ HEM-907 automated oscillometric sphygmomanometer after at least a 10-minute rest in a seated position, and the average of the last two measurements was used. Hypertension was defined by a systolic BP ≥ 140 mm Hg or a diastolic BP ≥ 90 mm Hg or antihypertensive drug treatment.

### Exclusion criteria

Participants were considered as eligible if they presented with T2D as defined previously. Participants were excluded if they had (a) no glucose measurement, or (b) missing information for any covariate. Exclusion criteria were applied for the baseline study (2003-06) and each follow-up period (2009-12, 2014-17 and 2018-21).

### Statistical analysis

Statistical analyses were performed using Stata version 18.0 for windows (Stata Corp, College Station, Texas, USA). Descriptive results were expressed as number of participants (percentage) or as average ± standard deviation. Bivariate analyses were performed using chi-square test for categorical variables and Student’s t-test for continuous variables. Multivariable analysis was performed using logistic regression and the results were expressed as Odds ratio (OR) and 95% confidence interval (CI). For control, two approaches were performed, using control level as the dependent variable: first, we assessed the associations for each individual antidiabetic drug (i.e. one antidiabetic drug = one model); second, we included all antidiabetic drugs in a single model. For newly diagnosed participants, we used each individual antidiabetic drug as the independent variable. All models were adjusted for age (continuous), marital status (alone, in couple), educational level (high, middle, low), smoking status (never, former, current), BMI categories (normal, overweight, obese), hypertension (yes, no) and presence of hypolipidemic drug treatment (yes, no). Statistical significance was assessed for a two-sided test with *p* < 0.05.

### Ethical statement

The institutional Ethics Committee of the University of Lausanne, which afterwards became the Ethics Commission of Canton Vaud (www.cer-vd.ch) approved the baseline CoLaus study (reference 16/03, decisions of 13th January and 10th February 2003). The approval was renewed for the first (reference 33/09, decision of 23rd February 2009), the second (reference 26/14, decision of 11th March 2014) and the third (reference PB_2018-00040, decision of 20th March 2018) follow-ups. The approval for the entire CoLaus|PsyCoLaus study was confirmed in 2021 (reference PB_2018-00038, 239/09, decision of 21st June 2021). The study was performed in agreement with the Helsinki declaration and its former amendments, and in accordance with the applicable Swiss legislation. All participants gave their signed informed consent before entering the study.

## Results

### Selection of participants

Of the initial 6733, 5064, 4881 and 3751 participants for the baseline, first, second and third follow-ups, 436 (6.5%), 539 (10.6%), 498 (10.2%) and 383 (10.2%) were considered as eligible and 434 (99.5%), 531 (98.5%), 347 (69.7%) and 255 (66.6%) were included, respectively. The reasons for exclusion (no glucose measurement, missing information for any covariate) are summarized in supplementary Fig. 1 and the comparison between included and excluded participants is summarized in supplementary Table 2. No consistent differences were found between excluded and included participants between the different surveys. In the second follow-up, included participants were less often female and were less likely to have hypertension compared to excluded participants. In the third follow-up, included participants were more often female (supplementary Table 2).

### Trends in antidiabetic drugs

**Fig. 1:**
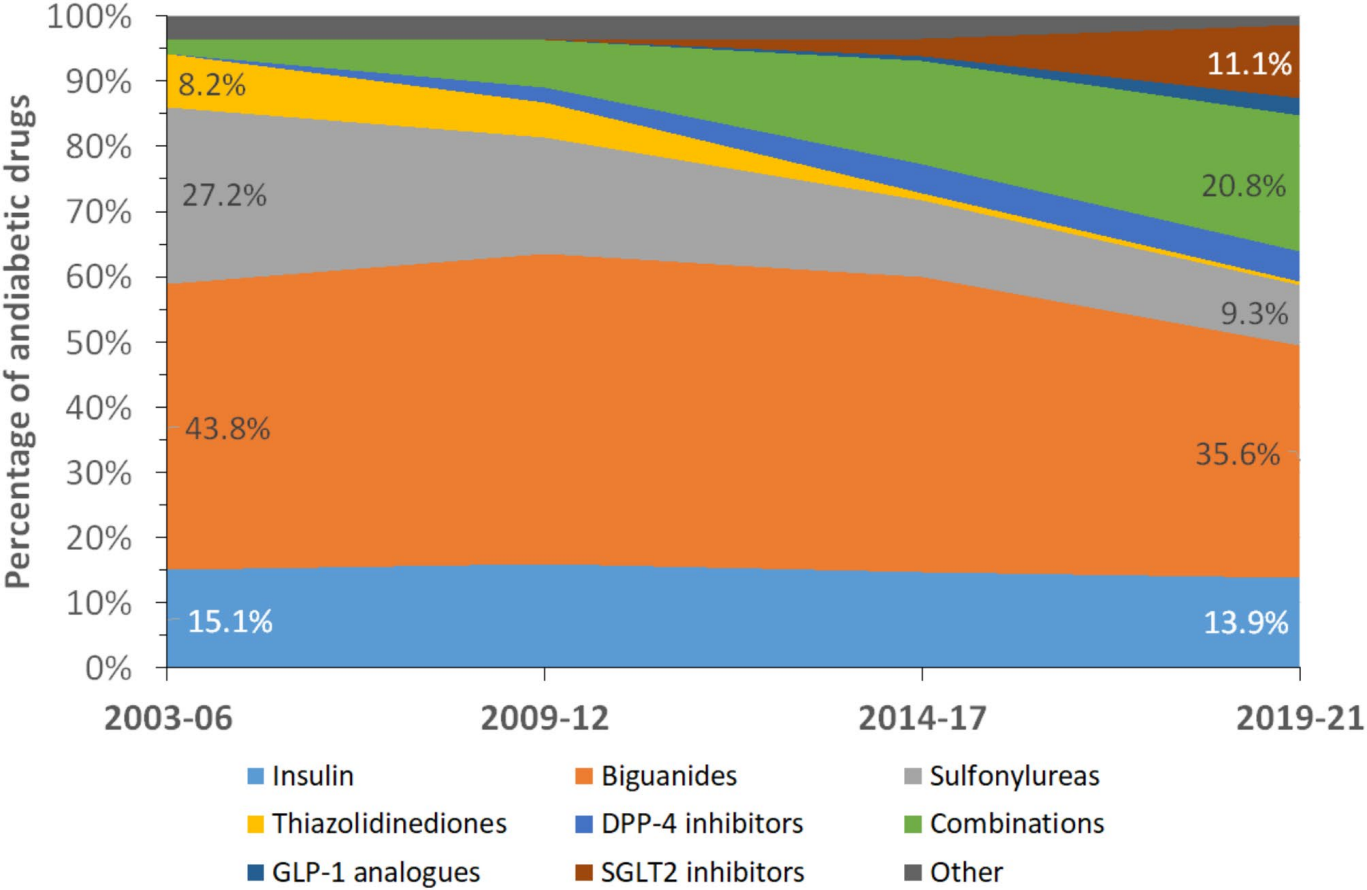
Trends in antidiabetic drug prescription, CoLaus study, Lausanne, Switzerland

**Fig. 2:**
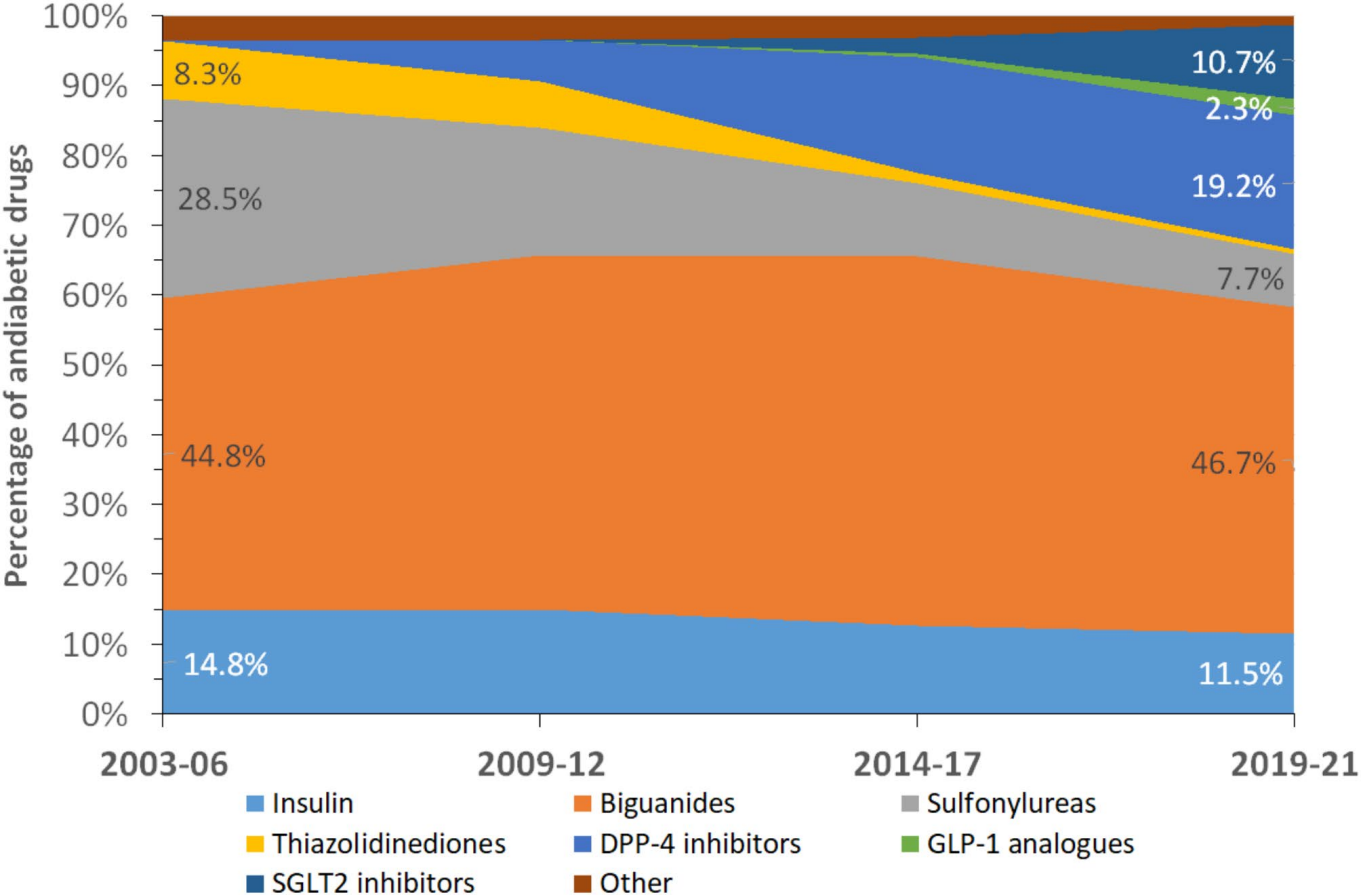
Trends in classes of antidiabetic drugs prescription, CoLaus study, Lausanne, Switzerland

The trends in the different antidiabetic drugs, expressed as % of total, are summarized in Fig. 1 for individual drugs and in Fig. 2 for antidiabetic drug classes. Between 2003 and 06 and 2019-21, the percentage of biguanides, sulfonylureas and thiazolidinediones decreased, while the percentage of oral drug combinations and SGLT2 inhibitors increased (Fig. 1). When oral drug combinations were split, the percentage of biguanides remained stable, the percentage of sulfonylureas and thiazolidinediones decreased, and the percentage of SGLT2 and DPP4 inhibitors increased (Fig. 2).

### Trends in diabetes management

Of the 434, 531, 347 and 255 included participants with diabetes in the baseline, first, second and third follow-ups, 274 (63.1%), 280 (52.7%), 268 (77.2%) and 195 (76.5%) were treated, respectively. The characteristics of participants according to diabetes treatment are summarized in supplementary Table 3. In all surveys except the third follow-up, treated participants were older than non-treated. At baseline, treated participants were more likely to have abdominal obesity or hypertension. In the first follow-up, treated participants were less often current smokers, had a higher BMI and were more likely to have hypertension. In the third follow-up, treated participants were more often female.

Of the 274, 280, 268 and 195 treated participants in the baseline, first, second and third follow-ups, 101 (36.9%), 103 (36.8%), 138 (51.5%) and 84 (43.1%) were controlled, respectively. The characteristics of participants according to diabetes control are summarized in Table 1. No consistent differences were found between participants with controlled and uncontrolled diabetes for all surveys. At baseline, controlled participants were more often female, while in the second follow-up, controlled participants were older. The associations between the classes of antidiabetic drugs and diabetes control are summarized in Table 2. At baseline, on bivariate analysis, controlled participants used fewer sulfonylureas; on multivariable analysis, use of sulfonylureas or of insulin was associated with a lower likelihood of being controlled. In the first follow up, on bivariate analysis, controlled participants used fewer DPP4 inhibitors, and use of DPP4 inhibitors was associated with a lower likelihood of being controlled. In the second follow-up, on bivariate analysis, there was a trend towards reduced use of DPP4 inhibitors among controlled participants, which became statistically significant after multivariable adjustment. In the third follow-up, there were no significant differences regarding antidiabetic drug classes according to diabetes control (Table 2). Similar conclusions were obtained when all antidiabetic drugs were simultaneously included in the model (supplementary Table 4).

**Table 1:** Characteristics of participants according to diabetes control, by survey period, CoLaus|PsyCoLaus study, Lausanne, Switzerland

	2003-06			2009-12			2014-17			2018-21		
	No	Yes	*P*-value	No	Yes	*P*-value	No	Yes	*P*-value	No	Yes	*P*-value
Sample size	173	101		177	103		130	138		111	84	
Women (%)	48 (27.8)	44 (43.6)	0.007	52 (29.4)	32 (31.1)	0.766	41 (31.5)	50 (36.2)	0.417	48 (43.2)	42 (50.0)	0.349
Age (years)	60.5 ± 9.0	60.9 ± 9.2	0.731	64.9 ± 8.7	66.2 ± 8.8	0.239	67.5 ± 9.1	69.9 ± 9.2	0.03	68.8 ± 9.0	68.4 ± 8.6	0.709
Education (%)			0.099			0.131			0.363			0.62
High	20 (11.6)	4 (4.0)		16 (9.0)	15 (14.6)		14 (10.8)	13 (9.4)		11 (9.9)	9 (10.7)	
Middle	36 (20.8)	22 (21.8)		36 (20.3)	27 (26.2)		34 (26.2)	27 (19.6)		32 (28.8)	19 (22.6)	
Low	117 (67.6)	75 (74.3)		125 (70.6)	61 (59.2)		82 (63.1)	98 (71.0)		68 (61.3)	56 (66.7)	
Living in couple (%)	114 (65.9)	66 (65.4)	0.926	103 (58.2)	64 (62.1)	0.517	86 (66.2)	83 (60.1)	0.308	62 (55.9)	50 (59.5)	0.608
Smoking status (%)			0.537			0.383			0.151			0.637
Never	51 (29.5)	36 (35.6)		61 (34.5)	29 (28.2)		36 (27.7)	49 (35.5)		47 (42.3)	30 (35.7)	
Former	82 (47.4)	42 (41.6)		88 (49.7)	60 (58.3)		71 (54.6)	59 (42.8)		47 (42.3)	39 (46.4)	
Current	40 (23.1)	23 (22.8)		28 (15.8)	14 (13.6)		23 (17.7)	30 (21.7)		17 (15.3)	15 (17.9)	
BMI (kg/m^2^)	31.0 ± 5.5	30.0 ± 5.9	0.16	31 ± 5.8	29.7 ± 4.5	0.059	30.0 ± 4.7	30.1 ± 5.0	0.949	29.9 ± 4.6	29.0 ± 5.5	0.241
BMI categories (%)			0.07			0.386			0.937			0.151
Normal	19 (11.0)	21 (20.8)		22 (12.7)	13 (12.9)		16 (12.4)	18 (13.3)		12 (11.1)	17 (20.5)	
Overweight	57 (33.0)	33 (32.7)		65 (37.6)	46 (45.5)		52 (40.3)	56 (41.5)		45 (41.7)	35 (42.2)	
Obese	97 (56.1)	47 (46.5)		86 (49.7)	42 (41.6)		61 (47.3)	61 (45.2)		51 (47.2)	31 (37.4)	
Abdominal obesity (%)	118 (68.2)	64 (63.4)	0.413	122 (70.9)	72 (69.9)	0.856	91 (70.5)	96 (71.6)	0.844	81 (73.6)	51 (61.5)	0.071
Hypertension (%)	142 (82.1)	79 (78.2)	0.435	142 (80.2)	82 (79.6)	0.901	103 (79.2)	111 (80.4)	0.806	95 (85.6)	63 (75.0)	0.062

BMI, body mass index. Results are expressed as number of participants (column percentage) for categorical variables and as average ± standard deviation for continuous variables. Between-group comparisons performed using chi-square for categorical variables and student’s t-test for continuous variables

**Table 2:** Distribution of antidiabetic drugs according to diabetes control, by survey period, CoLaus|PsyCoLaus study, Lausanne, Switzerland

	2003-06			2009-12			2014-17			2018-21		
	No	Yes	*P*-value	No	Yes	*P*-value	No	Yes	*P*-value	No	Yes	*P*-value
Sample size	173	101		177	103		130	138		111	84	
**Bivariate**												
Insulin	37 (21.4)	13 (12.9)	0.104	45 (25.4)	18 (17.5)	0.139	31 (23.9)	23 (16.7)	0.171	21 (18.9)	15 (17.9)	1
Biguanides	99 (57.2)	52 (51.5)	0.38	130 (73.5)	82 (79.6)	0.312	102 (78.5)	115 (83.3)	0.352	89 (80.2)	62 (73.8)	0.305
Sulfonylureas	71 (41.0)	25 (24.8)	0.008	53 (29.9)	23 (22.3)	0.21	26 (20.0)	16 (11.6)	0.066	17 (15.3)	8 (9.5)	0.282
Thiazolidinediones	19 (11.0)	9 (8.9)	0.682	20 (11.3)	7 (6.8)	0.294	2 (1.5)	5 (3.6)	0.448	1 (0.9)	1 (1.2)	1
DPP4 inhibitors	-	-		21 (11.9)	4 (3.9)	0.029	40 (30.8)	28 (20.3)	0.051	39 (35.1)	19 (22.6)	0.081
GLP1 analogues	-	-		-	-		1 (0.8)	1 (0.7)	1	4 (3.6)	3 (3.6)	1
SGLT2 inhibitors	-	-		-	-		6 (4.6)	5 (3.6)	0.764	21 (18.9)	9 (10.7)	0.16
Other	8 (4.6)	4 (4.0)	1	11 (6.2)	4 (3.9)	0.584	7 (5.4)	6 (4.4)	0.78	4 (3.6)	2 (2.4)	0.701
**Multivariable**												
Insulin	1 (ref)	0.46 (0.22–0.96)	0.038	1 (ref)	0.62 (0.32–1.18)	0.148	1 (ref)	0.66 (0.35–1.26)	0.208	1 (ref)	0.86 (0.40–1.86)	0.699
Biguanides	1 (ref)	0.90 (0.53–1.50)	0.675	1 (ref)	1.29 (0.68–2.44)	0.44	1 (ref)	1.44 (0.74–2.80)	0.284	1 (ref)	0.64 (0.31–1.34)	0.238
Sulfonylureas	1 (ref)	0.50 (0.28–0.89)	0.018	1 (ref)	0.65 (0.36–1.18)	0.157	1 (ref)	0.60 (0.29–1.22)	0.157	1 (ref)	0.61 (0.23–1.57)	0.304
Thiazolidinediones	1 (ref)	0.98 (0.40–2.37)	0.959	1 (ref)	0.52 (0.20–1.37)	0.188	1 (ref)	2.67 (0.48–15.0)	0.264	1 (ref)	1.25 (0.07–22.1)	0.881
DPP4 inhibitors	-	-		1 (ref)	0.27 (0.08–0.84)	0.024	1 (ref)	0.50 (0.28–0.90)	0.022	1 (ref)	0.57 (0.29–1.12)	0.103
GLP1 analogues	-	-		-	-		1 (ref)	1.04 (0.06–17.5)	0.977	1 (ref)	1.26 (0.26–6.17)	0.773
SGLT2 inhibitors	-	-		-	-		1 (ref)	0.87 (0.25–3.07)	0.827	1 (ref)	0.50 (0.21–1.22)	0.13
Other	1 (ref)	0.75 (0.21–2.69)	0.664	1 (ref)	0.56 (0.16–1.92)	0.353	1 (ref)	0.61 (0.18–2.09)	0.433	1 (ref)	0.64 (0.10–3.94)	0.632

-, no data. Results are expressed as number of participants (column percentage) for bivariate analyses and as odds ratio (95% confidence interval) for multivariable analyses. Bivariate analyses conducted using Fisher’s exact test and multivariable analyses conducted using logistic regression for each antidiabetic drug separately adjusting for age (continuous), marital status (alone, in couple), educational level (high, middle, low), smoking status (never, former, current), BMI categories (normal, overweight, obese), hypertension (yes, no) and presence of hypolipidemic drug treatment (yes, no)

When biguanides were prescribed, an association with DPP4 inhibitors was found in 9.4%, 26.3% and 33.8% of the cases in the first, second and third follow-ups, respectively. Only 1% and 3.3% of biguanides were associated with GLP1 analogues in the second and third follow-ups, respectively. Regarding SGLT2 inhibitors, an association was found in 4.2% and 8.6% of the cases in the second and third follow-ups, respectively.

### Management of newly diagnosed versus established diabetes

Overall, 151 new cases of diabetes were diagnosed and received antidiabetic drug treatment: 80 between the baseline and the first follow-up, 39 between the first and the second follow-ups, and 32 between the second and the third follow-ups. The characteristics of participants according to newly diagnosed or established diabetes are summarized in supplementary Table 5. In the first follow-up, participants with newly diagnosed diabetes were more often female, younger and non-smokers. In the following follow-ups, differences in smoking status were no longer statistically significant. Participants with newly diagnosed diabetes tended to be younger in the second and were more often female in the third follow-up.

The bivariate and multivariable analysis of antidiabetic drugs prescribed are summarized in Table 3. Participants with newly diagnosed diabetes were prescribed significantly less insulin, biguanides, sulfonylureas, thiazolidinediones and DPP4 inhibitors. On bivariate and multivariable analysis, participants with newly diagnosed diabetes had a higher likelihood of being adequately controlled than participants with established diabetes (Table 3).

**Table 3:** Antidiabetic drugs prescribed to old and newly diagnosed diabetes, CoLaus|PsyCoLaus study, Lausanne, Switzerland

		2009-12			2014-17			2018-21	
	Established	New	*P*-value	Established	New	*P*-value	Established	New	*P*-value
Sample size	200	80		229	39		163	32	
**Bivariate (%)**									
Insulin	60 (30.0)	3 (3.8)	< 0.001	52 (22.7)	2 (5.1)	0.009	36 (22.1)	0 (0)	0.001
Biguanides	143 (71.5)	69 (86.3)	0.009	186 (81.2)	31 (79.5)	0.826	130 (79.8)	21 (65.6)	0.104
Sulfonylureas	66 (33.0)	10 (12.5)	0.001	42 (18.3)	0 (0)	0.001	23 (14.1)	2 (6.3)	0.383
Thiazolidinediones	24 (12.0)	3 (3.8)	0.042	7 (3.1)	0 (0)	0.598	2 (1.2)	0 (0)	1.000
DPP4 inhibitors	20 (10.0)	5 (6.3)	0.365	64 (28.0)	4 (10.3)	0.017	54 (33.1)	4 (12.5)	0.020
GLP1 analogues	-	-		2 (0.9)	0 (0)	1.000	4 (2.5)	3 (9.4)	0.089
SGLT2 inhibitors	-	-		11 (4.8)	0 (0)	0.376	27 (16.6)	3 (9.4)	0.424
Other	13 (6.5)	2 (2.5)	0.246	12 (5.2)	1 (2.6)	0.699	6 (3.7)	0 (0)	0.592
Diabetes control	59 (29.5)	44 (55.0)	< 0.001	106 (46.3)	32 (82.1)	< 0.001	62 (38.0)	22 (68.8)	0.002
**Multivariable**									
Insulin	1 (ref.)	0.08 (0.02–0.29)	< 0.001	1 (ref.)	0.22 (0.05–0.95)	0.042	1 (ref.)	NE	
Biguanides	1 (ref.)	2.09 (0.97–4.47)	0.058	1 (ref.)	0.65 (0.25–1.64)	0.358	1 (ref.)	0.29 (0.11–0.76)	0.012
Sulfonylureas	1 (ref.)	0.29 (0.14–0.62)	0.001	1 (ref.)	NE		1 (ref.)	0.41 (0.08–2.01)	0.274
Thiazolidinediones	1 (ref.)	0.26 (0.07–0.95)	0.041	1 (ref.)	NE		1 (ref.)	NE	
DPP4 inhibitors	1 (ref.)	0.66 (0.22–1.92)	0.442	1 (ref.)	0.25 (0.08–0.76)	0.014	1 (ref.)	0.35 (0.11–1.09)	0.071
GLP1 analogues	-	-		1 (ref.)	NE		1 (ref.)	3.60 (0.58–22.5)	0.170
SGLT2 inhibitors	-	-		1 (ref.)	NE		1 (ref.)	0.58 (0.15–2.20)	0.424
Other	1 (ref.)	0.41 (0.08–2.05)	0.277	1 (ref.)	0.64 (0.07–5.63)	0.684	1 (ref.)	NE	
Diabetes control	1 (ref.)	3.39 (1.89–6.07)	< 0.001	1 (ref.)	5.41 (2.25–13.0)	< 0.001	1 (ref.)	3.47 (1.45–8.31)	0.005

-, no data; NE, not estimable. Results are expressed as number of participants (column percentage) for bivariate analyses and as odds ratio (95% confidence interval) for multivariable analyses. Bivariate analyses conducted using Fisher’s exact test and multivariable analyses conducted using logistic regression for each antidiabetic drug separately adjusting for age (continuous), marital status (alone, in couple), educational level (high, middle, low), smoking status (never, former, current), BMI categories (normal, overweight, obese), hypertension (yes, no) and presence of hypolipidemic drug treatment (yes, no)

## Discussion

Our results indicate that, despite the prescription of novel antidiabetic drugs, still half of participants treated for diabetes do not achieve adequate control. Our results also suggest that participants with newly diagnosed diabetes achieve much better control than participants with established diabetes.

### Trends in antidiabetic drugs

The decrease in sulfonylureas in our study is consistent with several other studies [11–15] and may be due to the growing awareness of hypoglycaemia and cardiovascular risk associated with these drugs [16]. Similarly, the decrease in thiazolidinediones in our study is consistent with other studies [11, 12, 14]. The slight decrease in insulin therapy in our study contrasts with increased use in other studies [12, 13] and might be due to difficulties in controlling diabetes, as indicated by the relatively low control rates consistently found in all surveys. Biguanides decreased (for individual drugs) or remained stable (for antidiabetic drug class) in our study, whereas they increased in other studies [11–15]. As most national and international guidelines propose metformin as a first-line drug treatment for T2D [6, 7], the reasons for this decrease are hard to explain. The increase of DPP4 or SGLT2 inhibitors and of GLP1 analogues in our study is consistent with several other studies [11–15], although in one study the use of DPP4 inhibitors tended to decrease [14]. Still, an increase in the association of biguanides with DPP4 inhibitors was found, which agrees with the international guidelines [7]. Conversely, the low association of GLP1 analogues with biguanides was unexpected; a possible explanation would be that SGLT2 inhibitors were preferred to GLP1 analogues, as both combinations (metformin + GLP1 analogues or metformin + SGLT2 inhibitors) are recommended by the Swiss Society for Endocrinology and Diabetes [6]. Overall, our results suggest that the Swiss guidelines tend to be implemented in clinical practice, although among a relatively small number of people.

Hence, and as observed elsewhere, our results indicate that in Switzerland, the prescription of sulfonylureas and thiazolidinediones is decreasing, being replaced by GLP1 analogues, SGLT2 and DPP4 inhibitors. Although the combination of DPP4 and SGLT2 inhibitors with metformin increased, the overall use of metformin- and insulin-based therapies decreased, in contrast to other studies [12, 13]. Differences in outcomes may stem from variations in clinical guidelines, prescribing practices, cost-effectiveness and regional healthcare priorities. Sociodemographic and patient-related factors, such as insulin resistance and comorbidities, also influence treatment choices.

### Trends in diabetes control

Despite the increasing availability of new antidiabetic drugs, there was little to no improvement in diabetes control, with one half of treated participants not achieving adequate FPG levels. Our findings are in agreement with the literature [4, 17–22]. Poor medication adherence contributes to lower diabetes control. A review of 71 studies estimated that only half (51.2%) of patients adhere to their medication [23]. Poorer diabetes control (higher HbA1c levels) has been shown to be associated with earlier intensification of treatment [24, 25]. The most poorly controlled diabetics are already under stronger medication, but their control may remain poor despite increased treatment.

### Management of newly diagnosed diabetes

Little is known how management of newly diagnosed diabetes and risk factor control have evolved over time. In our study, diet and BMI did not differ between newly diagnosed and established diabetics, suggesting that lifestyle changes, such as increased physical activity and weight loss, are not effectively implemented to control blood glucose levels. People newly diagnosed with diabetes may be more motivated to adhere to recommended medications, while those with long-term diabetes may relax their monitoring. Nevertheless, in a systematic review of 27 studies, duration of diabetes was not associated with medication adherence [26].

### Clinical implications

Maintaining long-term treatment for diabetes is essential to prevent cardiovascular complications [27] and poor medication adherence is associated with increased HbA1c levels, emergency department visits and hospitalizations [28, 29]. Side effects, mainly gastrointestinal and weight gain, hinder adherence to treatment [30]. Patients also lack information to better understand their condition, as well as the benefits and risks of treatment [31]. Our results show that, despite the availability of novel, potent antidiabetic drugs, no significant improvement in diabetes control was found. Hence, a closer monitoring of patients with diabetes should be performed, focusing on a healthy lifestyle, weight control, and adequate compliance to treatment.

Our results also show that participants with newly diagnosed diabetes had better blood glucose control. Clinicians should explore this window of opportunity to provide guidance regarding lifestyle changes and patient-centred care that addresses individual concerns and needs, to increase the likelihood of adequate diabetes control in the future. Metformin, as a first-line therapy for T2D, is widely endorsed by clinical guidelines due to its efficacy, safety profile, and cost-effectiveness [6, 7]. Deviations from these guidelines raise concerns about clinicians’ preferences, patient factors, or gaps in knowledge. Addressing non-adherence could improve patient outcomes by prioritizing evidence-based treatments, reducing complications, and optimizing resources.

Contrary to expected, participants on insulin or on the newer and more potent antidiabetic drugs such as GLP-1 analogues or SGLT2 inhibitors did not present with a better control of their condition. Possible explanations include a reverse causation, those drugs being prescribed to participants with difficulties in controlling their diabetes, or to the small number of participants receiving those drugs, leading to a low statistical power. Further, both DPP4 and SGLT2 inhibitors have a lower risk of hypoglycaemia and potential cardiovascular and renal protective effects, while their impact on glycaemic control may not be as potent as that of sulfonylureas, particularly in patients who are more insulin-resistant or those with advanced diabetes.

### Strengths and limitations

There are several strengths to this study. Firstly, the importance of different antidiabetic drugs in the management and control of diabetes in Switzerland was analysed over a period of eighteen years. Secondly, a wide range of socio-demographic covariates were analysed to better understand the facilitating and inhibiting factors for diabetes control.

There are also some limitations to this study. Firstly, it was conducted in a single location, and results might not apply to the entire country or to other settings. Still, the poor control of blood glucose levels is consistent with a previous study conducted in Geneva [22], and studies abroad [32, 33], suggesting that better control among patients with newly diagnosed diabetes might be a general trend. Secondly, the sample size was relatively small, which would have reduced the statistical power. It would be interesting to replicate our study in a larger sample. Thirdly, it was not possible to assess the exact posology of the antidiabetic drugs or the compliance of the participants towards their treatment. This could lead to a possible information bias, with some participants being considered as treated while not taking their medication. Fourthly, other comorbidities beyond hypertension, such as renal impairment, were not considered, yet they can impact the pharmacokinetics and efficacy of certain diabetes medications, influencing treatment outcomes. Fifthly, although the number of excluded participants was small in the first two surveys, it increased significantly in the other two. This might lead to an information bias if excluded participants had different treatment and control levels or received different antidiabetic medications than included ones. Still, as the characteristics of included and excluded participants were globally similar, it can be expected that those differences in management, if present, would be minor. Notwithstanding, it would be of importance if other studies could replicate our findings. Finally, we relied on FPG levels < 7 mmol/L to define diabetes control, which is a rather high level. Had we considered a lower level, the control rates would have been even lower than reported.

We conclude that, in a population-based sample of French-speaking Switzerland, at least half of participants treated for diabetes do not achieve adequate control, despite the availability of novel antidiabetic drugs. Participants with newly diagnosed diabetes achieve better control than participants with established diabetes.

## Electronic supplementary material

Below is the link to the electronic supplementary material.

Supplementary Material 1

Supplementary Material 2

## Data Availability

The data of CoLaus|PsyCoLaus study used in this article cannot be fully shared as they contain potentially sensitive personal information on participants. According to the Ethics Committee for Research of the Canton of Vaud, sharing these data would be a violation of the Swiss legislation with respect to privacy protection. However, coded individual-level data that do not allow researchers to identify participants are available upon request to researchers who meet the criteria for data sharing of the CoLaus|PsyCoLaus Datacenter (CHUV, Lausanne, Switzerland). Any researcher affiliated to a public or private research institution who complies with the CoLaus|PsyCoLaus standards can submit a research application to research.colaus@chuv.ch or research.psycolaus@chuv.ch. Proposals requiring baseline data only, will be evaluated by the baseline (local) Scientific Committee (SC) of the CoLaus and PsyCoLaus studies. Proposals requiring follow-up data will be evaluated by the follow-up (multicentric) SC of the CoLaus|PsyCoLaus cohort study. Detailed instructions for gaining access to the CoLaus|PsyCoLaus data used in this study are available at www.colaus-psycolaus.ch/professionals/how-to-collaborate/.
