# Supplementary figures and images for: Fifteen-year trends in diabetes drug management and control in French-speaking Switzerland
Source: Diabetol Metab Syndr. 2025 Feb 12;17:56. doi: 10.1186/s13098-025-01620-z (PMC11823013; doi:10.1186/s13098-025-01620-z)

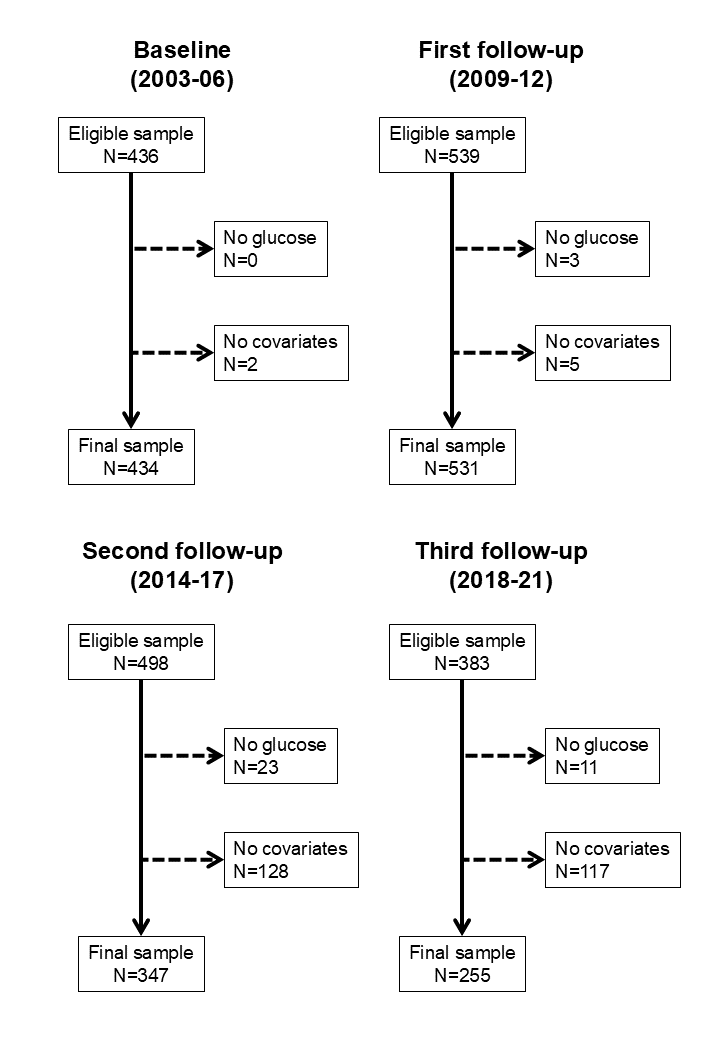

Supplement: Supplementary file 1 — Supplementary Material 1 [file 13098_2025_1620_MOESM1_ESM.png]
