# Supplementary material for: Fifteen-year trends in diabetes drug management and control in French-speaking Switzerland
Source: Diabetol Metab Syndr. 2025 Feb 12;17:56. doi: 10.1186/s13098-025-01620-z (PMC11823013; doi:10.1186/s13098-025-01620-z)
Supplement: Supplementary file 2 — Supplementary Material 2 [file 13098_2025_1620_MOESM2_ESM.docx]

# **Supplementary information**

**Supplementary table 1**: ATC codes of the antidiabetic drugs considered in this study

| **ATC code** | **Antidiabetic drug** |
| --- | --- |
| A10A | Insulins and analogues |
| A10BA | Biguanides |
| A10BB | Sulfonylureas |
| A10BC | Sulfonamides heterocyclic |
| A10BD | Combinations of oral blood glucose lowering drugs |
| A10BF | Alpha glucosidase inhibitors |
| A10BG | Thiazolidinediones |
| A10BH | DPP-4 inhibitors |
| A10BJ | GLP-1 analogues |
| A10BK | SGLT2 inhibitors |
| A10BX | Other blood glucose lowering drugs, excl. insulins |

ATC: anatomical, therapeutic, chemical. More information on <https://atcddd.fhi.no/atc_ddd_index/>

**Supplementary table 2**: characteristics of included and excluded participants, by survey period, CoLaus|PsyCoLaus study, Lausanne, Switzerland

|  |  | | **2003-06** |  | |  | | **2009-12** | | |  | |  | | **2014-17** | |  | |
| --- | --- | --- | --- | --- | --- | --- | --- | --- | --- | --- | --- | --- | --- | --- | --- | --- | --- | --- |
|  | **Included** | **Excluded** | | | **P-value** | | **Included** | | **Excluded** | **P-value** | | **Included** | | **Excluded** | | **P-value** | |  |
| Sample size | 434 | 2 | | |  | | 531 | | 8 |  | | 347 | | 151 | |  | |  |
| Women (%) | 141 (32.5) | 1 (50.0) | | | 0.546 | | 164 (30.9) | | 4 (50.0) | 0.264 | | 111 (32.0) | | 67 (44.4) | | 0.011 | |  |
| Age (years) | 59.6 ± 9.2 | 55.5 ± 12.7 | | | 0.524 | | 63.7 ± 9.2 | | 64.9 ± 10.2 | 0.709 | | 68.1 ± 9.4 | | 68.9 ± 9.1 | | 0.360 | |  |
| Education (%) |  |  | | | 0.549 | |  | |  | 0.741 | |  | |  | | 0.922 | |  |
| High | 44 (10.1) | 0 (0) | | |  | | 69 (13.0) | | 0 (0) |  | | 40 (11.5) | | 16 (10.7) | |  | |  |
| Middle | 98 (22.6) | 1 (50.0) | | |  | | 121 (22.8) | | 2 (28.6) |  | | 76 (21.9) | | 35 (23.3) | |  | |  |
| Low | 292 (67.3) | 1 (50.0) | | |  | | 341 (64.2) | | 5 (71.4) |  | | 231 (66.6) | | 99 (66.0) | |  | |  |
| Living in couple (%) | 281 (64.8) | 1 (100) | | | 1.000 | | 303 (57.1) | | 4 (50.0) | 0.731 | | 216 (62.3) | | 37 (52.9) | | 0.180 | |  |
| Smoking status (%) |  |  | | | 0.169 | |  | |  | 0.296 | |  | |  | | 0.262 | |  |
| Never | 143 (33.0) | 2 (100) | | |  | | 168 (31.6) | | 2 (40.0) |  | | 110 (31.7) | | 38 (40.9) | |  | |  |
| Former | 185 (42.6) | 0 (0) | | |  | | 255 (48.0) | | 1 (20.0) |  | | 169 (48.7) | | 39 (41.9) | |  | |  |
| Current | 106 (24.4) | 0 (0) | | |  | | 108 (20.3) | | 2 (40.0) |  | | 68 (19.6) | | 16 (17.2) | |  | |  |
| BMI (kg/m^2^) | 30.3 ± 5.7 | 24.5 ± 2.6 | | | 0.155 | | 30.0 ± 5.2 | | 30.4 ± 4.2 | 0.866 | | 30.0 ± 4.8 | | 30.5 ± 4.9 | | 0.310 | |  |
| BMI categories (%) |  |  | | | 0.145 | |  | |  | 1.000 | |  | |  | | 0.257 | |  |
| Normal | 72 (16.6) | 1 (50.0) | | |  | | 71 (13.7) | | 0 (0) |  | | 44 (12.9) | | 8 (7.8) | |  | |  |
| Overweight | 151 (34.8) | 1 (50.0) | | |  | | 227 (43.7) | | 3 (50.0) |  | | 141 (41.2) | | 50 (48.5) | |  | |  |
| Obese | 211 (48.6) | 0 (0) | | |  | | 222 (42.7) | | 3 (50.0) |  | | 157 (45.9) | | 45 (43.7) | |  | |  |
| Abdominal obesity (%) | 273 (62.9) | 0 (0) | | | 0.139 | | 352 (67.2) | | 5 (71.4) | 1.000 | | 241 (70.7) | | 76 (73.1) | | 0.711 | |  |
| Hypertension (%) | 331 (76.3) | 1 (50.0) | | | 0.421 | | 392 (73.8) | | 6 (75.0) | 1.000 | | 273 (78.7) | | 119 (88.2) | | 0.019 | |  |

BMI, body mass index. Results are expressed as number of participants (column percentage) for categorical variables and as average ± standard deviation for continuous variables. Between-group comparisons performed using Fisher’s exact test for categorical variables and student’s t-test for continuous variables. In the column excluded, the numbers might not add to the total due to missing information.

**Supplementary table 2 (continued)**: characteristics of included and excluded participants, by survey period, CoLaus|PsyCoLaus study, Lausanne, Switzerland

|  |  | | **2018-21** | |  | |
| --- | --- | --- | --- | --- | --- | --- |
|  | **Included** | **Excluded** | | **P-value** | |  |
| Sample size | 255 | 128 | |  | |  |
| Women (%) | 108 (42.4) | 36 (28.1) | | 0.007 | |  |
| Age (years) | 68.3 ± 8.8 | 69.1 ± 9.8 | | 0.428 | |  |
| Education (%) |  |  | | 0.123 | |  |
| High | 31 (12.2) | 20 (15.6) | |  | |  |
| Middle | 64 (25.1) | 21 (16.4) | |  | |  |
| Low | 160 (62.8) | 87 (68.0) | |  | |  |
| Living in couple (%) | 146 (57.3) | 35 (54.7) | | 0.778 | |  |
| Smoking status (%) |  |  | | 0.334 | |  |
| Never | 101 (39.6) | 25 (31.3) | |  | |  |
| Former | 115 (45.1) | 39 (48.8) | |  | |  |
| Current | 39 (15.3) | 16 (20.0) | |  | |  |
| BMI (kg/m^2^) | 29.6 ± 5.0 | 30.0 ± 4.6 | | 0.539 | |  |
| BMI categories (%) |  |  | | 0.737 | |  |
| Normal | 37 (14.7) | 12 (11.3) | |  | |  |
| Overweight | 106 (42.2) | 46 (43.4) | |  | |  |
| Obese | 108 (43.0) | 48 (45.3) | |  | |  |
| Abdominal obesity (%) | 172 (68.3) | 81 (77.1) | | 0.098 | |  |
| Hypertension (%) | 200 (78.4) | 102 (85.0) | | 0.162 | |  |

BMI, body mass index. Results are expressed as number of participants (column percentage) for categorical variables and as average ± standard deviation for continuous variables. Between-group comparisons performed using Fisher’s exact test for categorical variables and student’s t-test for continuous variables. In the column excluded, the numbers might not add to the total due to missing information.

**Supplementary table 3**: characteristics of participants according to presence or absence of antidiabetic drug treatment, by survey period, CoLaus|PsyCoLaus study, Lausanne, Switzerland

|  |  | **2003-06** | |  | |  | | **2009-12** | | |  | |  | | **2014-17** | |  | |
| --- | --- | --- | --- | --- | --- | --- | --- | --- | --- | --- | --- | --- | --- | --- | --- | --- | --- | --- |
|  | **No** | | **Yes** | | **P-value** | | **No** | | **Yes** | **P-value** | | **No** | | **Yes** | | **P-value** | |  |
| Sample size | 160 | | 274 | |  | | 251 | | 280 |  | | 79 | | 268 | |  | |  |
| Women (%) | 49 (30.6) | | 92 (33.6) | | 0.526 | | 80 (31.9) | | 84 (30.0) | 0.641 | | 20 (25.3) | | 91 (34.0) | | 0.148 | |  |
| Age (years) | 57.9 ± 9.2 | | 60.7 ± 9.0 | | 0.002 | | 61.8 ± 9.4 | | 65.3 ± 8.8 | <0.001 | | 65.8 ± 9.6 | | 68.8 ± 9.2 | | 0.014 | |  |
| Education (%) |  | |  | | 0.233 | |  | |  | 0.340 | |  | |  | | 0.271 | |  |
| High | 20 (12.5) | | 24 (8.8) | |  | | 38 (15.1) | | 31 (11.1) |  | | 13 (16.5) | | 27 (10.1) | |  | |  |
| Middle | 40 (25.0) | | 58 (21.2) | |  | | 58 (23.1) | | 63 (22.5) |  | | 15 (19.0) | | 61 (22.8) | |  | |  |
| Low | 100 (62.5) | | 192 (70.1) | |  | | 155 (61.8) | | 186 (66.4) |  | | 51 (64.6) | | 180 (67.2) | |  | |  |
| Living in couple (%) | 101 (63.1) | | 180 (65.7) | | 0.589 | | 136 (54.2) | | 167 (59.6) | 0.204 | | 47 (59.5) | | 169 (63.1) | | 0.566 | |  |
| Smoking status (%) |  | |  | | 0.341 | |  | |  | 0.004 | |  | |  | | 0.986 | |  |
| Never | 56 (35.0) | | 87 (31.8) | |  | | 78 (31.1) | | 90 (32.1) |  | | 25 (31.7) | | 85 (31.7) | |  | |  |
| Former | 61 (38.1) | | 124 (45.3) | |  | | 107 (42.6) | | 148 (52.9) |  | | 39 (49.4) | | 130 (48.5) | |  | |  |
| Current | 43 (26.9) | | 63 (23.0) | |  | | 66 (26.3) | | 42 (15.0) |  | | 15 (19.0) | | 53 (19.8) | |  | |  |
| BMI (kg/m^2^) | 29.7 ± 5.7 | | 30.6 ± 5.6 | | 0.113 | | 29.4 ± 4.8 | | 30.5 ± 5.4 | 0.021 | | 29.8 ± 4.7 | | 30.1 ± 4.8 | | 0.677 | |  |
| BMI categories (%) |  | |  | | 0.084 | |  | |  | 0.147 | |  | |  | | 0.974 | |  |
| Normal | 32 (20.0) | | 40 (14.6) | |  | | 36 (14.6) | | 35 (12.8) |  | | 10 (12.8) | | 34 (12.9) | |  | |  |
| Overweight | 61 (38.1) | | 90 (32.9) | |  | | 116 (47.2) | | 111 (40.5) |  | | 33 (42.3) | | 108 (40.9) | |  | |  |
| Obese | 67 (41.9) | | 144 (52.6) | |  | | 94 (38.2) | | 128 (46.7) |  | | 35 (44.9) | | 122 (46.2) | |  | |  |
| Abdominal obesity (%) | 91 (56.9) | | 182 (66.4) | | 0.047 | | 158 (63.5) | | 194 (70.6) | 0.084 | | 54 (69.2) | | 187 (71.1) | | 0.750 | |  |
| Hypertension (%) | 110 (68.8) | | 221 (80.7) | | 0.005 | | 168 (66.9) | | 224 (80) | 0.001 | | 59 (74.7) | | 214 (79.9) | | 0.324 | |  |

BMI, body mass index. Results are expressed as number of participants (column percentage) for categorical variables and as average ± standard deviation for continuous variables. Between-group comparisons performed using chi-square for categorical variables and student’s t-test for continuous variables.

**Supplementary table 3 (continued)**: characteristics of participants according to presence or absence of antidiabetic drug treatment, by survey period, CoLaus|PsyCoLaus study, Lausanne, Switzerland

|  |  | **2018-21** | |  | |  |
| --- | --- | --- | --- | --- | --- | --- |
|  | **No** | | **Yes** | | **P-value** | |
| Sample size | 60 | | 195 | |  | |
| Women (%) | 18 (30.0) | | 90 (46.2) | | 0.027 | |
| Age (years) | 67.2 ± 8.6 | | 68.6 ± 8.8 | | 0.260 | |
| Education (%) |  | |  | | 0.232 | |
| High | 11 (18.3) | | 20 (10.3) | |  | |
| Middle | 13 (21.7) | | 51 (26.2) | |  | |
| Low | 36 (60.0) | | 124 (63.6) | |  | |
| Living in couple (%) | 34 (56.7) | | 112 (57.4) | | 0.916 | |
| Smoking status (%) |  | |  | | 0.650 | |
| Never | 24 (40.0) | | 77 (39.5) | |  | |
| Former | 29 (48.3) | | 86 (44.1) | |  | |
| Current | 7 (11.7) | | 32 (16.4) | |  | |
| BMI (kg/m^2^) | 30.2 ± 4.8 | | 29.5 ± 5.0 | | 0.359 | |
| BMI categories (%) |  | |  | | 0.937 | |
| Normal | 8 (13.3) | | 29 (15.2) | |  | |
| Overweight | 26 (43.3) | | 80 (41.9) | |  | |
| Obese | 26 (43.3) | | 82 (42.9) | |  | |
| Abdominal obesity (%) | 40 (67.8) | | 132 (68.4) | | 0.931 | |
| Hypertension (%) | 42 (70.0) | | 158 (81.0) | | 0.069 | |

BMI, body mass index. Results are expressed as number of participants (column percentage) for categorical variables and as average ± standard deviation for continuous variables. Between-group comparisons performed using chi-square for categorical variables and student’s t-test for continuous variables.

**Supplementary table 4**: multivariable analysis of the joint distribution of antidiabetic drugs according to diabetes control, by survey period, CoLaus|PsyCoLaus study, Lausanne, Switzerland

|  |  | **2003-06** |  |  | **2009-12** |  |  | **2014-17** |  |
| --- | --- | --- | --- | --- | --- | --- | --- | --- | --- |
|  | **No** | **Yes** | **P-value** | **No** | **Yes** | **P-value** | **No** | **Yes** | **P-value** |
| Sample size | 173 | 101 |  | 177 | 103 |  | 130 | 138 |  |
| Insulin | 1 (ref) | 0.34 (0.16 - 0.75) | 0.007 | 1 (ref) | 0.58 (0.29 - 1.18) | 0.132 | 1 (ref) | 0.67 (0.34 - 1.33) | 0.255 |
| Biguanides | 1 (ref) | 0.76 (0.44 - 1.31) | 0.318 | 1 (ref) | 0.96 (0.47 - 1.97) | 0.914 | 1 (ref) | 1.28 (0.63 - 2.61) | 0.491 |
| Sulfonylureas | 1 (ref) | 0.41 (0.23 - 0.75) | 0.004 | 1 (ref) | 0.68 (0.35 - 1.30) | 0.244 | 1 (ref) | 0.53 (0.25 - 1.14) | 0.105 |
| Thiazolidinediones | 1 (ref) | 0.95 (0.38 - 2.36) | 0.913 | 1 (ref) | 0.55 (0.20 - 1.50) | 0.243 | 1 (ref) | 3.65 (0.59 - 22.5) | 0.162 |
| DPP4 inhibitors | - | - |  | 1 (ref) | 0.28 (0.09 - 0.88) | 0.029 | 1 (ref) | 0.47 (0.26 - 0.86) | 0.014 |
| GLP1 analogues | - | - |  | - | - |  | 1 (ref) | 1.81 (0.09 - 36.6) | 0.697 |
| SGLT2 inhibitors | - | - |  | - | - |  | 1 (ref) | 0.85 (0.21 - 3.42) | 0.822 |
| Other | 1 (ref) | 0.65 (0.18 - 2.40) | 0.519 | 1 (ref) | 0.57 (0.16 - 1.99) | 0.378 | 1 (ref) | 0.72 (0.20 - 2.56) | 0.611 |

-, no data. Results are expressed as number of participants (column percentage) for bivariate analyses and as odds ratio (95% confidence interval) for multivariable analyses. Bivariate analyses conducted using Fisher’s exact test and multivariable analyses conducted using logistic regression using all antidiabetic drugs adjusting for age (continuous), marital status (alone, in couple), educational level (high, middle, low), smoking status (never, former, current), BMI categories (normal, overweight, obese), hypertension (yes, no) and presence of hypolipidemic drug treatment (yes, no).

**Supplementary table 4 (continued)**: multivariable analysis of the joint distribution of antidiabetic drugs according to diabetes control, by survey period, CoLaus|PsyCoLaus study, Lausanne, Switzerland

|  |  | **2018-21** |  |
| --- | --- | --- | --- |
|  | **No** | **Yes** | **P-value** |
| Sample size | 111 | 84 |  |
| Insulin | 1 (ref) | 0.83 (0.35 - 1.97) | 0.677 |
| Biguanides | 1 (ref) | 0.47 (0.20 - 1.13) | 0.091 |
| Sulfonylureas | 1 (ref) | 0.59 (0.21 - 1.66) | 0.317 |
| Thiazolidinediones | 1 (ref) | 1.18 (0.06 - 21.8) | 0.913 |
| DPP4 inhibitors | 1 (ref) | 0.63 (0.31 - 1.29) | 0.211 |
| GLP1 analogues | 1 (ref) | 1.24 (0.23 - 6.53) | 0.802 |
| SGLT2 inhibitors | 1 (ref) | 0.40 (0.15 - 1.07) | 0.068 |
| Other | 1 (ref) | 0.96 (0.13 - 7.13) | 0.966 |

-, no data. Results are expressed as number of participants (column percentage) for bivariate analyses and as odds ratio (95% confidence interval) for multivariable analyses. Bivariate analyses conducted using Fisher’s exact test and multivariable analyses conducted using logistic regression using all antidiabetic drugs adjusting for age (continuous), marital status (alone, in couple), educational level (high, middle, low), smoking status (never, former, current), BMI categories (normal, overweight, obese), hypertension (yes, no) and presence of hypolipidemic drug treatment (yes, no).

**Supplementary table 5**: characteristics of participants according to newly diagnosed or established diabetes, by survey period, CoLaus|PsyCoLaus study, Lausanne, Switzerland

|  |  | **2009-12** |  |  | **2014-17** |  |  | **2018-21** |  |
| --- | --- | --- | --- | --- | --- | --- | --- | --- | --- |
|  | **Established** | **New** | **P-value** | **Established** | **New** | **P-value** | **Established** | **New** | **P-value** |
| Sample size | 200 | 80 |  | 229 | 39 |  | 163 | 32 |  |
| Women (%) | 53 (26.5) | 31 (38.8) | 0.043 | 73 (31.9) | 18 (46.2) | 0.082 | 69 (42.3) | 21 (65.6) | 0.016 |
| Age (years) | 66.1 ± 8.3 | 63.5 ± 9.7 | 0.024 | 69.2 ± 9.3 | 66.1 ± 8.6 | 0.050 | 68.9 ± 8.9 | 67.1 ± 8.4 | 0.291 |
| Education (%) |  |  | 0.607 |  |  | 0.534 |  |  | 0.386 |
| High | 21 (10.5) | 10 (12.5) |  | 25 (10.9) | 2 (5.1) |  | 15 (9.2) | 5 (15.6) |  |
| Middle | 48 (24.0) | 15 (18.8) |  | 52 (22.7) | 9 (23.1) |  | 45 (27.6) | 6 (18.8) |  |
| Low | 131 (65.5) | 55 (68.8) |  | 152 (66.4) | 28 (71.8) |  | 103 (63.2) | 21 (65.6) |  |
| Living in couple (%) | 122 (61.0) | 45 (56.3) | 0.464 | 145 (63.3) | 24 (61.5) | 0.831 | 95 (58.3) | 17 (53.1) | 0.590 |
| Smoking status (%) |  |  | 0.042 |  |  | 0.550 |  |  | 0.343 |
| Never | 58 (29.0) | 32 (40.0) |  | 70 (30.6) | 15 (38.5) |  | 65 (39.9) | 12 (37.5) |  |
| Former | 106 (53.0) | 42 (52.5) |  | 114 (49.8) | 16 (41.0) |  | 74 (45.4) | 12 (37.5) |  |
| Current | 36 (18.0) | 6 (7.5) |  | 45 (19.7) | 8 (20.5) |  | 24 (14.7) | 8 (25.0) |  |
| BMI (kg/m^2^) | 30.4 ± 5.6 | 30.7 ± 5.0 | 0.617 | 30.0 ± 4.7 | 30.5 ± 5.8 | 0.517 | 29.2 ± 4.7 | 31.0 ± 6.5 | 0.060 |
| BMI categories (%) |  |  | 0.109 |  |  | 0.127 |  |  | 0.259 |
| Normal | 30 (15.5) | 5 (6.3) |  | 26 (11.6) | 8 (20.5) |  | 24 (15.0) | 5 (16.1) |  |
| Overweight | 75 (38.7) | 36 (45.0) |  | 97 (43.1) | 11 (28.2) |  | 71 (44.4) | 9 (29.0) |  |
| Obese | 89 (45.9) | 39 (48.8) |  | 102 (45.3) | 20 (51.3) |  | 65 (40.6) | 17 (54.8) |  |
| Abdominal obesity (%) | 136 (69.4) | 58 (73.4) | 0.507 | 161 (71.9) | 26 (66.7) | 0.508 | 107 (66.1) | 25 (80.7) | 0.109 |
| Hypertension (%) | 164 (82.0) | 60 (75.0) | 0.186 | 185 (80.8) | 29 (74.4) | 0.355 | 133 (81.6) | 25 (78.1) | 0.647 |
| Antidiabetic diet (%) | 112 (56.0) | 42 (52.5) | 0.595 | 76 (33.2) | 10 (25.6) | 0.351 | 73 (44.8) | 9 (28.1) | 0.081 |
| Any diet (%) | 133 (66.5) | 51 (63.8) | 0.661 | 101 (44.1) | 15 (38.5) | 0.511 | 90 (55.2) | 13 (40.6) | 0.131 |
| Sedentary status (%) § | 109 (78.4) | 45 (76.3) | 0.740 | 89 (70.1) | 24 (80.0) | 0.277 | NA | NA |  |

BMI, body mass index; NA, not available. § only available for 198 (2009-2012) and 157 (2014-2017) participants. Results are expressed as number of participants (column percentage) for categorical variables and as average ± standard deviation for continuous variables. Between-group comparisons performed using chi-square for categorical variables and student’s t-test for continuous variables.
